# Foliar Moisture Content from the Spectral Signature for Wildfire Risk Assessments in Valparaíso-Chile

**DOI:** 10.3390/s19245475

**Published:** 2019-12-12

**Authors:** Juan Villacrés, Tito Arevalo-Ramirez, Andrés Fuentes, Pedro Reszka, Fernando Auat Cheein

**Affiliations:** 1 Department of Electronic Engineering, Universidad Técnica Federico Santa María, Valparaiso 2390123, Chile; juan.villacres@sansano.usm.cl (J.V.); tito.arevalo@sansano.usm.cl (T.A.-R.); 2 Department of Industrial Engineering, Universidad Técnica Federico Santa María, Valparaiso 2390123, Chile; andres.fuentes@usm.cl; 3 Faculty of Engineering and Sciences, Universidad Adolfo Ibáñez, Santiago 7941169, Chile; pedro.reszka@uai.cl

**Keywords:** fuel moisture content, wildland urban interface, leaves spectral signature

## Abstract

Fuel moisture content (FMC) proved to be one of the most relevant parameters for controlling fire behavior and risk, particularly at the wildland-urban interface (WUI). Data relating FMC to spectral indexes for different species are an important requirement identified by the wildfire safety community. In Valparaíso, the WUI is mainly composed of *Eucalyptus Globulus* and *Pinus Radiata*—commonly found in Mediterranean WUI areas—which represent the 97.51% of the forests plantation inventory. In this work we study the spectral signature of these species under different levels of FMC. In particular, we analyze the behavior of the spectral reflectance per each species at five dehydration stages, obtaining eighteen spectral indexes related to water content and, for *Eucalyptus Globulus*, the area of each leave—associated with the water content—is also computed. As the main outcome of this research, we provide a validated linear regression model associated with each spectral index and the fuel moisture content and moisture loss, per each species studied.

## 1. Introduction

**Figure 1:**
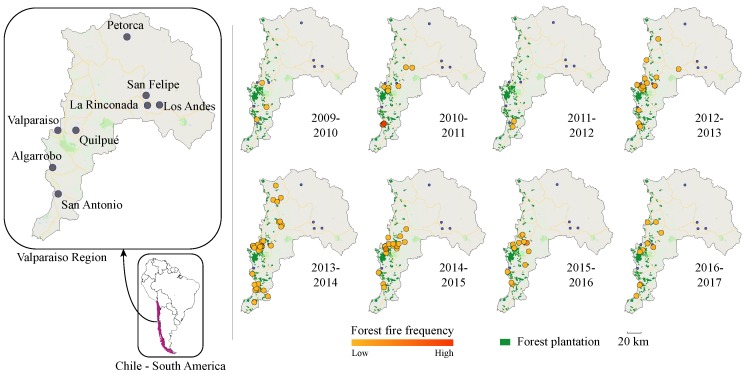
Forest plantation and forest fire frequency in Valparaíso Region, Chile. The forest fire frequency is obtained from CONAF database (free available at [7]); the forest plantation is adapted from CONAMA and CONAF [35].

The latest wildfire seasons in Chile have caused significant human, ecological and economic losses in cities, in the forestry industry and in protected areas. Recently, the fires in early 2017 caused 11 fatalities, burned more than 550,000 ha and destroyed more than 1000 houses [1]. In particular, Valparaíso—a central region in Chile—has suffered the greatest losses from wildfires and represents the most challenging scenario for wildfire prevention, detection, and response, due to its topographical and urban social features [2]. According to Chile’s Forest Service (CONAF), from 2003 to 2018, 12,868 recorded wildfires burned 121,328.81 ha in Valparaiso [3]. The prone of Valparaíso to wildfires is given by the introduction of flammable species—i.e., *Eucalyptus Globulus*, *Pinus Radiata*—[3,4], both species represent the 97.51% of the forest plantation [5]. Moreover, approximately 90% of such wildfires have been related to anthropogenic activities including transit of vehicles or aircraft, recreational activities, power line failures and arson attacks [6]. The sequence of maps presented in Figure 1 is a timeline of forest fire frequency in Valparaíso. Such figure offers a spatial visualization of areas majority affected [7]. Thus, given the susceptibility of Valparaíso to wildfires, it becomes crucial to allocate the areas with a higher risk for an adequate forest and wildland–urban interfaces (WUI) management.

Risk-based tools for decision making to manage forests and the WUI are cost-effective solutions to allocate resources in areas with higher risks [8,9]. However, their use requires an adequate wildfire modeling [10]. Such modeling needs of a reliable knowledge of the processes occurring in the solid and gaseous phases during the combustion of wildland fuels. Their ignition is an outstanding problem in fire and combustion science, and is far from being solved [11]. Furthermore, the wind speed, the fuel moisture content (FMC) and the weight of the fuel bed are some factors on which the wildfire modeling propagation depends [12]. In particular, the fine FMC (live and dead fuels) showed acceptable evidence of producing good results in the modeling of real-world fire-spread rate [13].

Fuel Moisture Content, which represents the amount of water contained relative to the amount of vegetation dry mass, can be measured or estimated from field samplings, gravimetric methods, and spectral measurements. The first two methods achieve high accuracy [14], but their results are not extensible at local, regional, and global scales [15]; being the last one the most suitable to larger extension, if the FMC is retrieved from satellite imagery as in [16]. The FMC estimation from optical sensors data is primarily made by empirical (statistical) or physical techniques [16]. The former approach establishes a statistical connection between an objective parameter—obtained in field measurements—and reflectance or vegetation indexes (VIs) [17]. Several VIs have been developed to estimate water content from the regions of the electromagnetic spectrum (e.g., visible, near–infrared, and shortwave infrared) [18,19,20,21,22,23,24,25,26,27,28,29,30,31]. On the other hand, the physical models—i.e., Radiative Transfer Models (RTM)—are independent of the site or the species. Therefore, RTMs may be used to construct “universal” VIs to retrieve vegetative parameters [32]. However, the spectra simulated from RTMs might be unrealistic if some criteria are not included in its parametrization as mentioned in [33].

**Figure 2:**
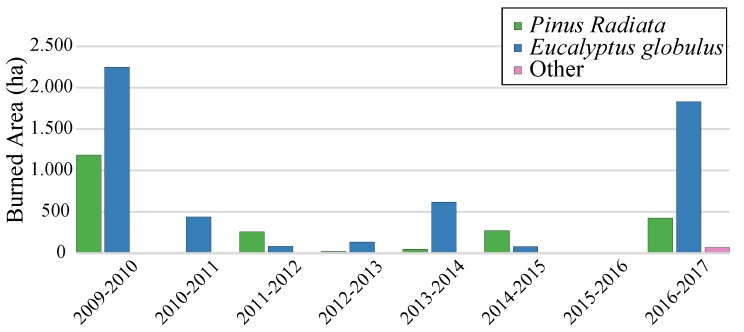
Hectares burned of Valparaiso’s forest plantation during 2009–2017 [34].

In this work, we analyze the water content of *Eucalyptus Globulus* and *Pinus Radiata* using their spectral signature, motivated by the lack of information about these two species in the WUI of Valparaiso. As expected, being dominant species in the forest plantation inventory, the area burned in recent years is more representative in both species, as shown in Figure 2 [34]. For each species, a total of 90 samples were used to measure the spectral reflectance and the mass at different stages of dehydration and senescence. A novel aspect of this work is that we present an evolution of several spectral indexes used in remote sensing for the estimation of biomass moisture content as a function of drying time and residual moisture content. It is expected that the methodologies and the data we present will be a contribution to the wildfire community working on remote sensing tools. Finally, this work is part of the project *Understanding Wildfire Hazards Posed by Ignition in Continuous and Discontinuous Configurations*, funded by CONICYT (the Chilean National Science Foundation) in an attempt to understand the wildfire risks of Valparaiso region in Chile.

## 2. Materials and Methods

The field sampling was carried out from August 2018 to March 2019. Leaf samples were collected from Valparaíso WUI, corresponding to *Eucalyptus Globulus* and *Pinus Radiata*. The samples were collected on days without precipitation during the previous 24 h. To maintain the freshness of the leaves, branches were cut and stored in plastic bags as recommended in [36]. Then, in less than one hour, the leaves were separated individually from the branches [37,38,39,40]. In the case of eucalyptus, the measurements were made for each leaf, while for pine, approximately 5 grams of pine needles were used, due to the difficulty of registering the spectral signature of a single needle with the sensors used in this study. A total of 90 samples were employed for each species. Once the fresh leaves were cut, the mass was obtained with a 0.1 mg resolution Kern PFB 120-3 precision balance. Leaf spectral reflectance was measured with a high-resolution ASD mineral spectrometer with a spectral range of 350–2500 nm. To record the spectral reflectance, the contact probe of the ASD spectrometer was placed flush against the sample using a white background (white spectralon). The samples were illuminated using the contact probe with a tungsten filament. The spectrometer calibration was performed every 20 readings to ensure the accuracy of the measurements. The reflectance of the white calibration pattern (white spectralon) was used by the spectrometer to set the base line up and calibrate itself [41].

**Figure 3:**
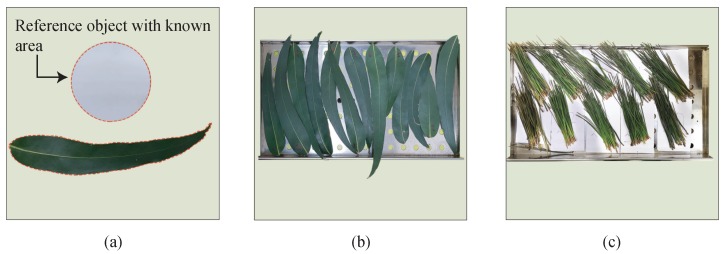
Leaves disposition before dehydration process. (**a**) shows the reference object used to estimate leaves’ area; (**b**,**c**) show the leaves on the tray as captured by the camera.

To estimate the area of the eucalyptus leaves, a reference object with a known area was used. A camera (12 MP, f/1.7 aperture with a focal length of 26 mm) was placed twenty centimeters above both samples, to photograph the entire area of the leaf and the reference object. Then, the estimation of the leaf area is calculated as the ratio of the pixels belonging to the leaf and the pixels in the reference object with a known area as shown in Figure 3a.

Next, the fresh leaves were placed in a Memmert UN30 drying oven at 65 ${}^{\circ }$C for a fixed time. Figure 3b,c show how the samples were placed in the tray before the dehydration process. Such dehydration temperature was chosen since when assessing the flammability of wildland fuels, it must be ensured that the fuel being analyzed has not lost any flammable content (i.e., that it has not pyrolyzed) so as not to affect the combustion behavior of the samples. The common practice of all combustion scientists working on wildland fuel fire behavior is to dry the samples between 60 and 80 ${}^{\circ }$C [42,43,44,45,46,47,48,49]. In the case of eucalyptus, the time was set to 15 min, while for pines the time was 60 min (such time intervals were chosen following the guidelines published in [50,51]). The dehydration process was repeated three times. Then, the leaves were placed in the oven for 24 h at 65 ${}^{\circ }$C to obtain constant mass (dry mass) [37,40,52]. Finally, the dry leaves were weighed and the reflectance spectrum was recorded. A detailed description of the instruments used and their specifications are shown in Table 1.

### Water Content and Vegetation Indexes

Based on the mass of the leaves at different dehydration stages, the water content is expressed as Fuel Moisture Content (FMC) with fresh—${}_{f}$—or dry basis—${}_{d}$—[39,52,53,54] and Equivalent Water Thickness (EWT) [52,53,55,56,57,58]. The former depends only on the leaf mass (Equations (1) and (2)), while the latter also requires its area (Equation (3)):

(1) $$\begin{array}{c} {\mathrm{FMC}}_{f}(\%)=\frac{W_{f,t}-W_{d}}{W_{f,t}}\times 100\% \end{array}$$

(2) $$\begin{array}{c} {\mathrm{FMC}}_{d}(\%)=\frac{W_{f,t}-W_{d}}{W_{d}}\times 100\% \end{array}$$

(3) $$\begin{array}{cc} \mathrm{EWT}\left(\frac{g}{{cm}^{2}}\right) & =\frac{W_{f,t}-W_{d}}{A} \end{array}$$

where $W_{f,t}$ (g) is the leaf fresh weight, *t* is the time in the oven, $W_{d}$ (g) is the leaf dry weight (after 24 h at 65 ${}^{\circ }$C) and *A* (cm${}^{2}$) is the leaf area. To avoid misinterpretations, we refer to FMC${}_{d}$ just as FMC. As just presented, the water content is obtained during an invasive and time-consuming process of dehydration.

The water content estimation from the spectral signature has been widely studied for a broad variety of vegetation species. Based on the review of the literature, we selected eighteen vegetation indexes that appear frequently in studies related to the use of VIs for the water content estimation (see Table 2 for VIs nomenclatures, references, and a brief description [59,60,61,62,63,64,65,66,67,68,69,70]). For example, in [59] the MSI, NDWI, TM5/TM7, and WI were used to estimate the leaf FMC, and EWT from remotely sensed reflectance. On the other hand, the leaf water status was estimated using the NDWI and the leaf water content index (LWCI) to monitor the forest fire risk [60]. In [61] it is presented the estimation of EWT in eucalyptus leaves using MSI, TM5/TM7, WI, NDWI, and Normalized Difference Vegetation Index (NDVI). However, such eucalyptus species was not *Eucalyptus Globulus*, and the leaves were not dehydrated. Based on field spectroscopy data, the EWT was estimated using the following indexes WI, SRWI, NDWI, fWBI, SIWSI, and NDII [62]. Also, the same indexes were compared against the use of full-spectrum and continuum removal for leaf-level EWT retrieval in commercial vineyards [63]. In cotton leaves, the EWT and FMC were estimated employing NDII, NDWI1, NDWI2, WI, WBI, fWBI, SRWI, SRWI1, SRWI2, MSI1, MSI2, and SIWSI [64].

## 3. Results

**Figure 4:**
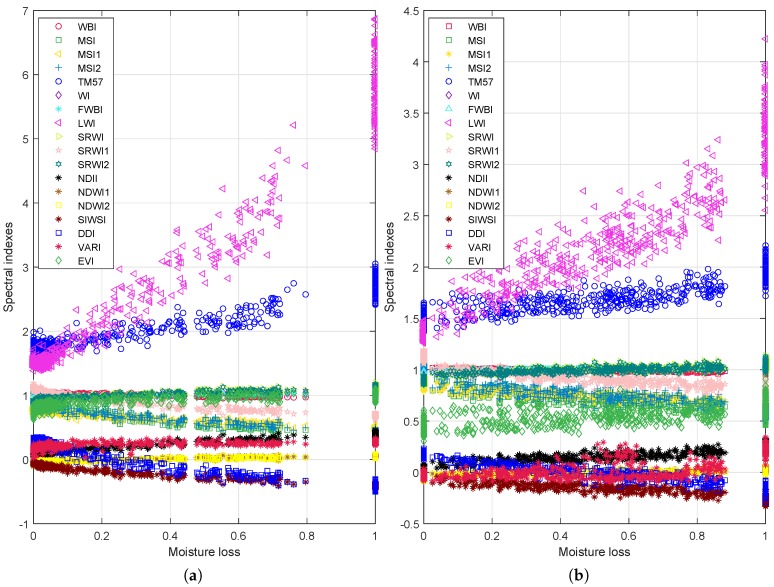
Relationship between moisture loss and the vegetation indexes. (**a**) shows the results for the *Eucalyptus Globulus* case, and (**b**) for the *Pinus Radiata* case.

**Figure 5:**
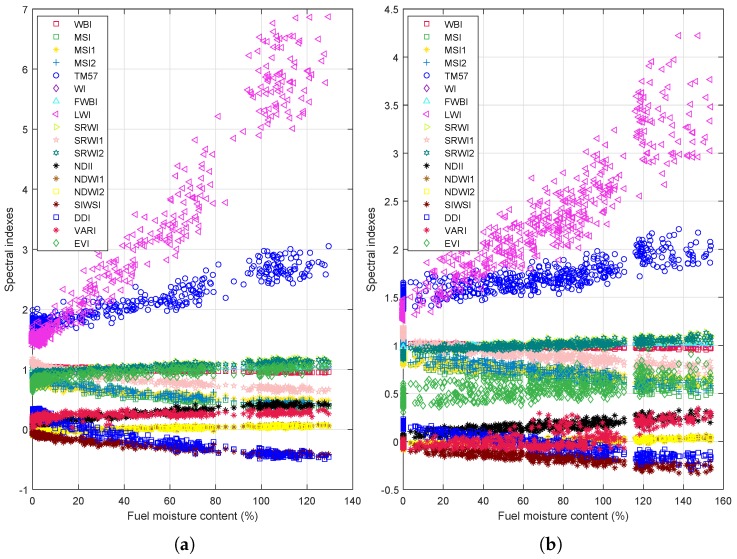
Relationship between fuel moisture content (FMC) and the vegetation indexes. The FMC with dry basis is calculated according to Equation (1). (**a**) shows the results for the *Eucalyptus Globulus* case, whereas (**b**) shows the results for the *Pinus Radiata* case.

**Figure 6:**
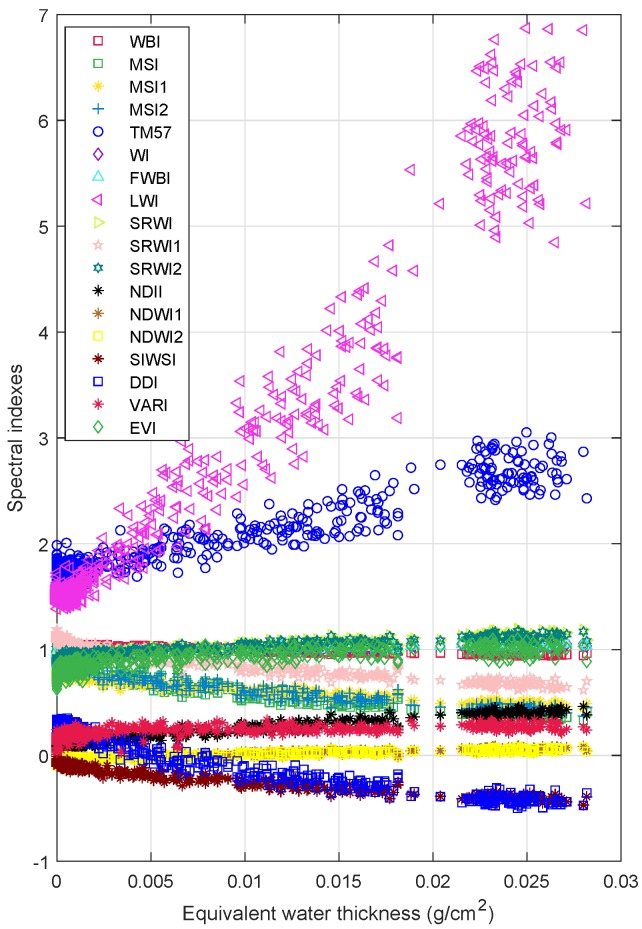
Relationship between equivalent water thickness (EWT) and *Eucalyptus Globulus* vegetation indexes. The EWT is calculated according to Equation (3).

**Figure A1:**
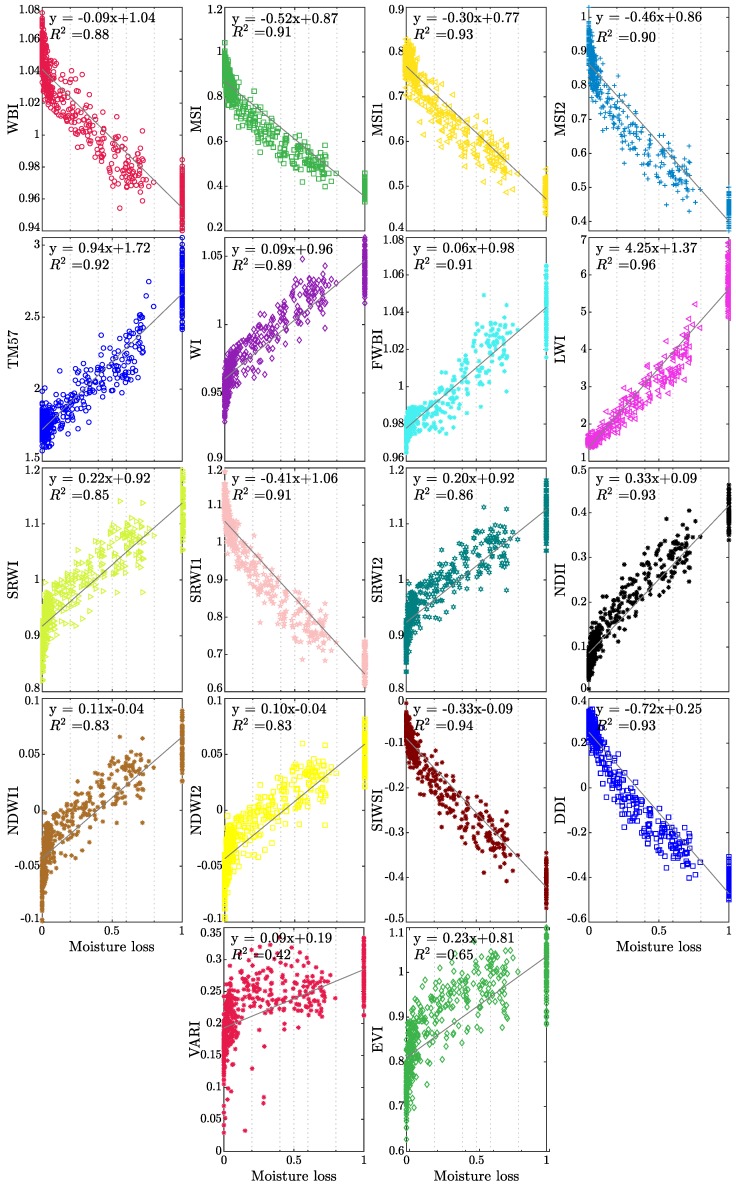
Linear regression of the moisture loss and the vegetation indexes for the *Eucalyptus Globulus*.

**Figure A2:**
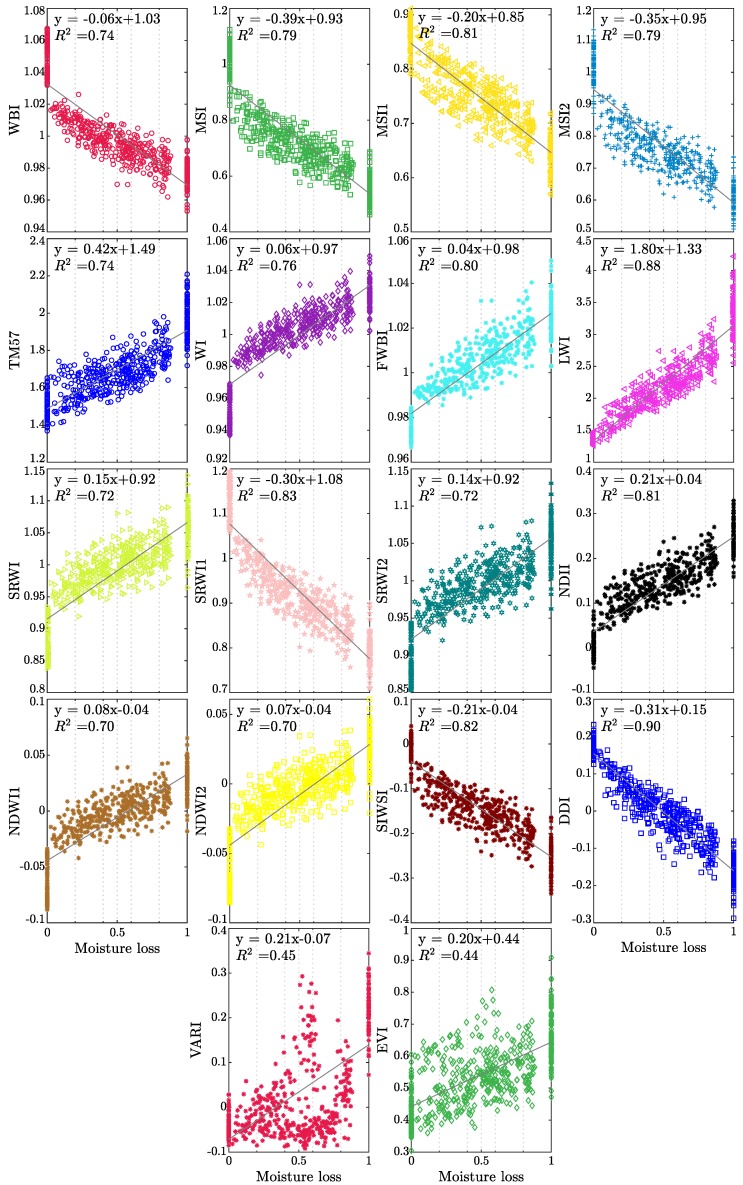
Linear regression of the moisture loss and the vegetation indexes for the *Pinus Radiata*.

**Figure A3:**
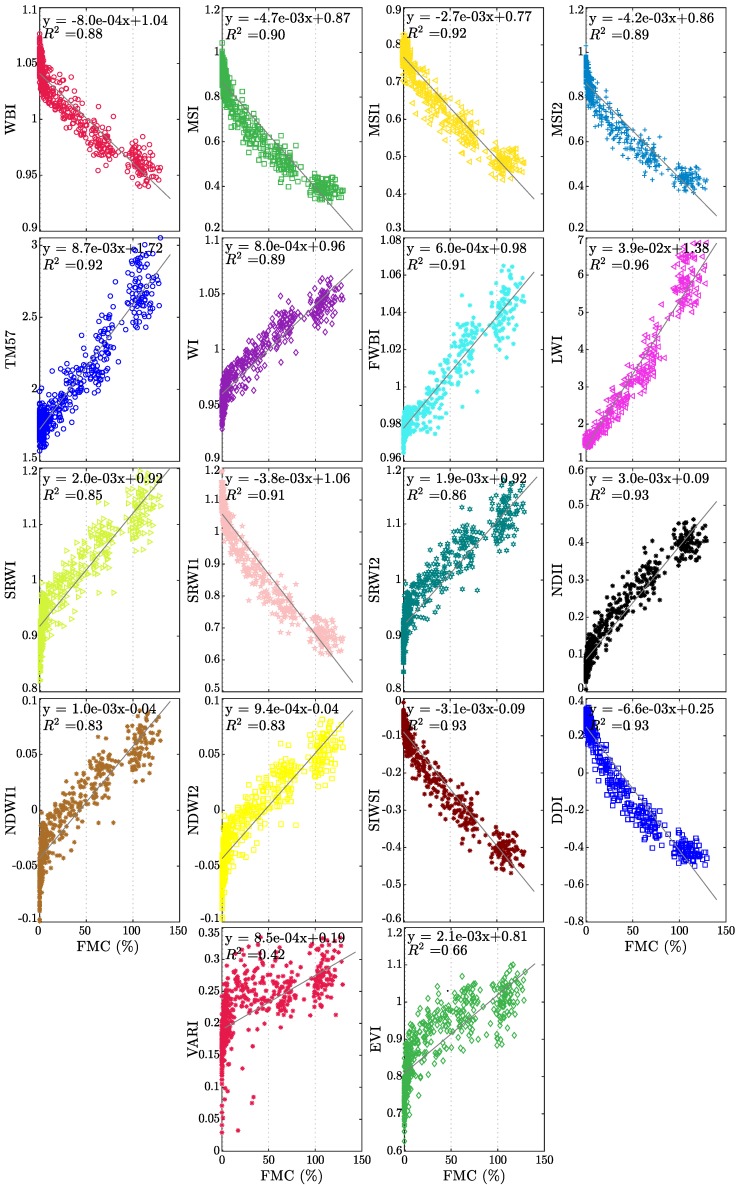
Linear regression of the fuel moisture content and the vegetation indexes for the *Eucalyptus Globulus*.

**Figure A4:**
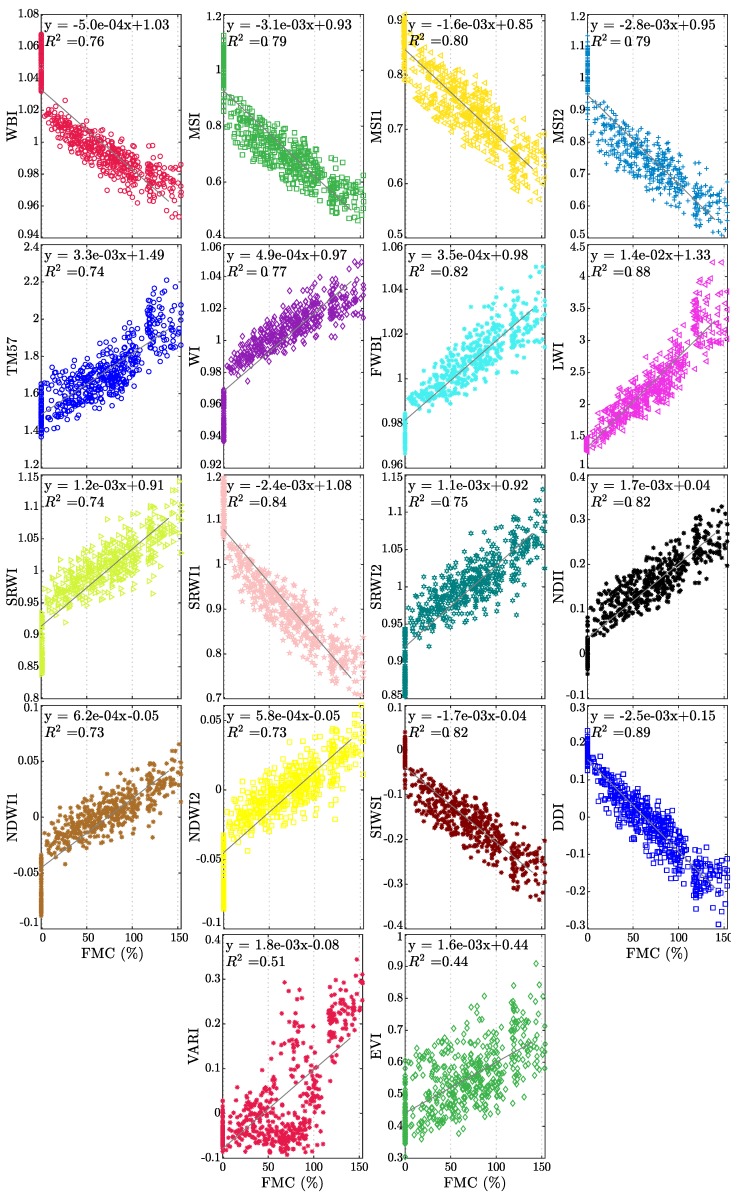
Linear regression of the fuel moisture content and the vegetation indexes for the *Pinus Radiata*.

**Figure A5:**
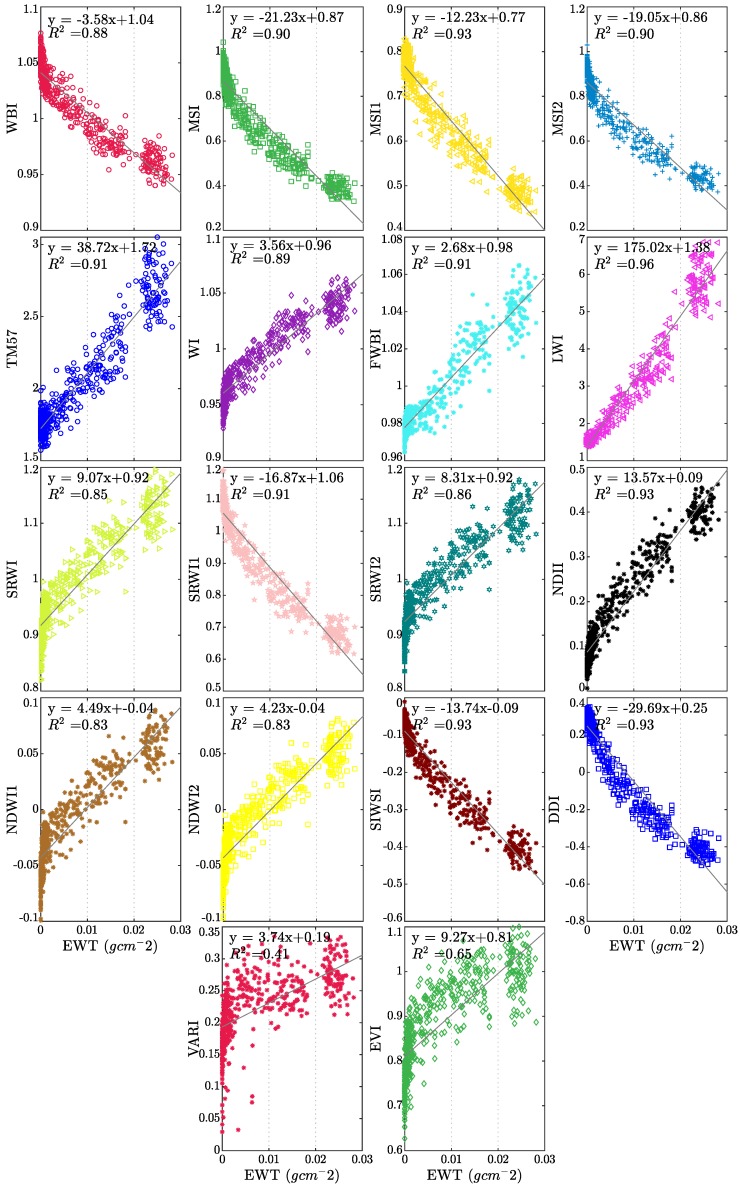
Linear regression of the equivalent water thickness for the *Eucalyptus Globulus* case.

The evolution of the eighteen spectral indexes corresponding to moisture loss for *Eucalyptus Globulus* and *Pinus Radiata* is shown in Figure 4. The moisture loss is normalized as the ratio of water contained in each stage of dehydration over the total amount of water. That is, the 0 value means that the leaves do not contain water (after 24 h of drying), meanwhile, the moisture loss 1 represents that the leaves are fresh (recently collected). Figure 5 shows the evolution of the selected vegetation indexes as a function of fuel moisture content (Equation (2)) for eucalyptus and pines. Values of FMC bigger than 100% implies that more than 50% of the leaves’ mass is water. Specifically, the maximum FMC contained in eucalyptus leaves is 129%, whereas the maximum FMC in pine needles is 153%. Finally, the variation of the spectral indexes when the equivalent water thickness of eucalyptus changes is presented in Figure 6. As can be seen in Figure 4, Figure 5 and Figure 6, the dehydration process allows us to have a wide range of values of moisture loss, FMC, and EWT. In addition, the behavior of the eighteen vegetation indexes is proportional to the water indicators. The aforementioned behavior is modeled as a linear regression in Appendix A (Figure A1, Figure A2, Figure A3, Figure A4, Figure A5), where a individual linear model is presented with its corresponding coefficient of determination $R^{2}$.

## 4. Discussion

Figure 4, Figure 5 and Figure 6 suggest that the relationship between the leaf water content (expressed in terms of: moisture loss, FMC, and EWT) and spectral indexes, for both species, is linear. In particular Figure A1 and Figure A2 evidence that all spectral indexes appeared to behave linearly with a $R^{2}$ of 0.41 in the worst case—VARI index for EWT estimation in eucalyptus—and of 0.96 in the best case—LWI index for moisture loss and EWT for eucalyptus—These values are similar to values founded in previous works [22,24,26,27,29,75], yet these works assessed the water content of others species.

The indexes with the lowest $R^{2}$ values—VARI 0.41 for EWT and EVI 0.44 for FMC and moisture loss—could advise that the index behavior is not linear. Nonetheless, the main reason can be associated with the dispersion of index values; the fitted model does not consider their variance. Specifically, the indexes derived from *Pinus Radiata* leaves present the biggest variations, as can be appreciated in Figure A2 and Figure A4. The variance of the index values shows that the *Pinus Radiata* leaves did not dry uniformly because of their initial water content and arrangement in the trail (sets of 5 g). Nevertheless, despite these issues, the robustness of the indexes can be inferred based on the variance and $R^{2}$ value. In particular, the indexes that showed the greater $R^{2}$ values are LWI and DDI, DDI achieved the greater $R^{2}$ value. Therefore, the behavior of spectral indexes and linear regression advise that the most suitable index to assess the water content in acicular leaves (*Pinus Radiata* leaves) is DDI.

Regarding to *Eucalyptus Globulus* leaves, the linear relationship between water content (moisture loss, FMC, and EWT)) and spectral indexes is more evident for this specie, reaching an $R^{2}$ value of 0.96, see Figure A1, Figure A3 and Figure A5. Moreover, the distribution of index values is narrower than the values obtained for *Pinus Radiata*. This suggest that the samples arrangement (see Figure 3) and drying process performed better than for non acicular leaves.

For both species (*Eucalyptus Globulus*, *Pinus Radiata*), the initial conditions of leaves can explain the index values dispersion. When the leaves were taken, it was assumed that all leaves samples had the same water status. Nevertheless, this is not always the case since the samples were taken randomly from several branches on different locations and environmental conditions (the only constraint considered was the absence of rain at least 24 h before taking the samples). Thus, the initial water status for the samples was not all the same. Then, at each dehydration stage, the leaves samples did not dry at the same level. On the other side, it is to be noted that we have not performed yet a correlation between the geographical location of the samples, the climate conditions, water status (FMC, EWT) and the eighteen vegetation indexes obtained.

In brief, despite the index values dispersion issue, results showed that water status in leaves can be assessed in different dehydration stages by the reflectance of leaves.

## 5. Conclusions

This work has shown the results of analyzing the spectral signature (at different dehydration levels) of the two most prevalent species in Valparaíso region, Chile, within the research project *Understanding Wildfire Hazards Posed by Ignition in Continuous and Discontinuous Configurations*, funded by CONICYT (the Chilean National Science Foundation), as an attempt to understand wildfire risks. The two species studied were *Eucalyptus Globulus* and *Pinus Radiata*, which represent the 97.5% of the vegetation. The samples were randomly collected from different locations and eighteen vegetation indexes—namely: WBI, MSI, MSI1, MSI2, TM5/TM7, WI, fWBI, LWI, SRWI, SRWI1, SRWI2, NDII, NDWI1, NDWI2, SIWSI, DDI, VARI, and EVI—were determined. We found that such indexes behave linearly under moisture loss and fuel moisture content estimation. In particular, LWI and DDI index have performed the best linear behavior for each species (*Eucalyptus Globulus*, *Pinus Radiata*), respectively. LWI reach an $R^{2}=0.96$ in all water status characterizations (moisture, FMC, and EWT), and DDI achieved a $R^{2}=0.90$ for moisture loss and $R^{2}=0.89$ for FMC. Moreover, these indexes showed the lowest dispersion. Therefore, the assessment of wildfire risk behavior can be further enhanced by the behavior of each spectral index showed in the present work and can be extended to other research fields, such as agriculture.

## Figures and Tables

**Table 1:** Technical Specifications of the instruments used in the procedure.

Instrument	Technical Specifications
TerraSpec 4 Hi-Res Mineral spectrometer	Wavelength range: 350–2500 nm
	Resolution: 3 nm at 700 nm and 6 nm at 1400/2100 nm
	Reproducibility: 0.1 nm
	Accuracy: 0.5 nm
Balance Kern PFB 120-3	Readability: 0.001 g
	Maximum capacity: 120 g
Universal Oven Memmert UN30	Temperature: −5 ${}^{\circ }$C and +300 ${}^{\circ }$C respect the environmental temperature
	Temperature control: Digital PID

**Table 2:** Vegetation Indexes related with the foliar moisture content. The equation column is represented by $R_{x}$, where *R* is the reflectance and ${}_{x}$ the wavelength.

Spectral Indexes	Equations
Water Band Index (WBI) is a good indicator of water status when the Relative Water Content (RWC) is smaller than 80–85 percent [18].	$R_{970}/R_{900}$
Moisture Stress Index (MSI) is correlated with the liquid water and MSI should be correlated with the Leaf Area Index (LAI) of a leaf [19].	$R_{1600}/R_{820}$
Moisture Stress Index 1 (MSI1) were derived from the TMS bands simple ratio. These indexes were used to estimate forest damage that can be attributed to moisture and anatomy of the vegetation [21].	$R_{1650}/R_{1230}$
Moisture Stress Index 2 (MSI2) Similar to the MSI1 index [21].	$R_{1650}/R_{830}$
Ratio of Thematic Mapper Band 5 to Band 7 (TM5/TM7) were used to estimate the density of vegetation through the Leaf Water Content (LWC) [22].	$R_{1650}/R_{2220}$
Water Index (WI) is correlated with a wide range of plant water concentration (FMC) obtained through a severe dehydration [23].	$R_{900}/R_{970}$
Floating-position Water Band Index (fWBI) was obtained from the relation $R_{900}$ and the minimum value in the range $R_{930}$ and $R_{980}$. This index was correlated with the area-weighted content of vegetation under stress conditions [71].	$\frac{R_{900}}{\min\left(R_{930}-R_{980}\right)}$
Leaf Water Index (LWI) exhibited a strong correlation with RWC in a laboratory standpoint, but it is not suitable for field measurement due to the influence of the atmospheric effects [26].	$R_{1300}/R_{1450}$
Simple Ratio Water Index (SRWI) was studied as a linking between leaf and canopy models with LWC [24].	$R_{860}/R_{1240}$
Simple Ratio Water Index 1(SRWI1) Simple Ratio Water Index 1 and 2 were obtained after a study of the water status in vineyards. These indexes showed a correlation with EWT and FMC (fresh and dry basis) [27].	$R_{1350}/R_{870}$
Simple Ratio Water Index 2 (SRWI2) similar to SRWI1 [27].	$R_{880}/R_{1265}$
Normalized Difference Infrared Index (NDII) is correlated with canopy water status. NDII was developed using the wavelengths that match the bands 3, 4 and 5 of Landsat-D Thematic Mapper [29].	$\frac{\left(R_{850}-R_{1650}\right)}{\left(R_{850}+R_{1650}\right)}$
Normalized difference Water Index 1 (NDWI1) is based in two narrow channels of the Landsat TM and it is sensitive to changes in the EWT [25].	$\frac{\left(R_{860}-R_{1240}\right)}{\left(R_{860}+R_{1240}\right)}$
Normalized difference Water Index 2 (NDWI2) is correlated with water content indicators (specially with EWT) at leaf level [27].	$\frac{\left(R_{870}-R_{1260}\right)}{\left(R_{870}+R_{1260}\right)}$
Shortwave Infrared Water Stress (SIWSI) was developed as indicator of water stress in a semiarid environment [30].	$\frac{\left(R_{1640}-R_{858}\right)}{\left(R_{1640}+R_{858}\right)}$
Double Difference Index (DDI) was presented to estimate the chlorophyll in leaves [72]. However, this index showed a strong correlation with EWT in a large simulated database [28].	$2R_{1530}-R_{1005}-R_{2055}$
Visible Atmospheric Resistant Index (VARI) is a sensitive indicator of the vegetation fraction (VF) from levels moderate to high [31]. Nonetheless, this index has been used for FMC estimation [73,74,75].	$\frac{\left(R_{green}-R_{red}\right)}{\left(R_{green}+R_{red}-R_{blue}\right)}$
Enhanced Vegetation Index (EVI) is an index derived from MODIS bands, it includes terms for atmosphere resistance and soil adjustment [76].	$2.5\frac{\left(R_{nir}-R_{red}\right)}{\left(R_{nir}+6R_{red}-7.5R_{blue}+1\right)}$
